# Optimal feedback correction in string quartet synchronization

**DOI:** 10.1098/rsif.2013.1125

**Published:** 2014-04-06

**Authors:** Alan M. Wing, Satoshi Endo, Adrian Bradbury, Dirk Vorberg

**Affiliations:** 1 SyMoN Lab, School of Psychology, University of Birmingham, Birmingham B152TT, UK; 2 Institute for Information-Oriented Control, Technische Universität München, München 80333, Germany; 3 Royal Academy of Music, University of London, Marylebone Road, London NW15HT, UK; 4 Institut für Psychologie, Westfälische Wilhelms-Universität Münster, Fliednerstrasse 21, Münster 48149, Germany

**Keywords:** music ensemble, synchronization, feedback correction, expressive variation, time-series models

## Abstract

Control of relative timing is critical in ensemble music performance. We hypothesize that players respond to and correct asynchronies in tone onsets that arise from fluctuations in their individual tempos. We propose a first-order linear phase correction model and demonstrate that optimal performance that minimizes asynchrony variance predicts a specific value for the correction gain. In two separate case studies, two internationally recognized string quartets repeatedly performed a short excerpt from the fourth movement of Haydn's quartet Op. 74 no. 1, with intentional, but unrehearsed, expressive variations in timing. Time series analysis of successive tone onset asynchronies was used to estimate correction gains for all pairs of players. On average, both quartets exhibited near-optimal gain. However, individual gains revealed contrasting patterns of adjustment between some pairs of players. In one quartet, the first violinist exhibited less adjustment to the others compared with their adjustment to her. In the second quartet, the levels of correction by the first violinist matched those exhibited by the others. These correction patterns may be seen as reflecting contrasting strategies of first-violin-led autocracy versus democracy. The time series approach we propose affords a sensitive method for investigating subtle contrasts in music ensemble synchronization.

## 1. Introduction

Coordination of movements to an external rhythmic auditory stimulus is a widespread biological phenomenon that occurs in non-human animals [1], as well as in humans from an early age [2], and possibly relates to vocal mimicry abilities [1,3]. Social groups frequently engage in activities which involve coordination of timing between group members. In many such activities, success depends on tightly synchronized timing. Moreover, engaging in coordinated timing activity has been shown to strengthen group cohesion [4]. In some cases, for example in rowing eights, timing is not the goal of the endeavour, yet each individual participant's timing is still closely linked to the timing of the group [5]. In other cases, for instance music performance, timing is an explicit goal of the activity [6]. In these examples, the question arises: how do participants in a group adjust their timing to each other? In this paper, we propose a feedback correction model of timing in ensemble music performance. The model includes correction gain terms within each pair of players; we show that, on average, players approximate an optimum, defined as the minimal variance of asynchrony, albeit with notable exceptions that reflect on established musical practice.

Ensemble musical performance involves control of timing, both within and between players. While individual players are expected to time successive tone onsets according to the tempo and notation of the written score, successful performance as a group also requires each player to control the timing of his or her tone onsets relative to those of the other players. Such relative timing is likely to be particularly important to the listener. Thus, by extrapolation of Weber's law, asynchronies of tens of milliseconds in the onsets of tones that are intended to sound together might be expected to be much more apparent than differences of tens of milliseconds in intervals lasting several hundreds of milliseconds between successive tones [7].

In general, players do not time tone onsets exactly as written in the score. As part of expressive interpretation of musical works, they tend to introduce departures from the scored timing [8]. Such local departures from overall tempo place demands on the control of relative timing—the phase of the tones produced by the player introducing the timing departure is changed relative to the phases of the other players. One aspect of rehearsal is thus to agree on expressive variations, so that players introduce timing departures together and maintain relative timing [9,10]. However, even with agreement on the musical interpretation of the piece in place, individual players may still choose to vary the timing of tone onsets in certain passages from one performance to the next [9–11]. Thus, David Soyer, cellist in the Guarneri Quartet, commenting on *rubato* (departures from scored tempo) observed: ‘We try … to avoid impositions (of rhythm and tempo). If one player takes a little musical liberty, the quartet goes along with him. We allow each other freedom—but there's a natural give and take … A moment of *ritardando* or *rubato* should not sound contrived (by being planned); it should be allowed to happen naturally’ [12, p. 16]. In other words, quartet players of professional standard are prepared to adjust their individual timings in order to restore relative phase with one another and so maintain overall ensemble as the performance evolves.

Timing in musical performance may also be subjected to a degree of unintended variation, for instance due to rhythmic complexity, technical demands of other dimensions of performance such as pitch or loudness, attention lapses and because biological timing is also inherently variable [13]. While prolonged individual practice may be expected to minimize such unintended variation, it is unlikely to remove it completely. Thus, both expressive variation and unintended fluctuation may affect the timing of a given player's tone onsets. So, it is reasonable to suppose that between-player adjustment of relative timing is important in achieving synchronized ensemble performance.

Anecdotal evidence of between-player timing adjustment has been presented for four-handed piano by Shaffer [14] and for violin and viola in a string quartet by Moore & Chen [15]. Both studies report a degree of asynchrony between the two players, which, although variable, appears to be regulated as there is no evidence of progressive divergence in timing of tone onsets of the two players, as would be expected if they were performing independently. A quantitative study of between-player timing adjustment was carried out by Goebl & Palmer [16]. The measure they used was the ‘inter-tone interval’ (ITI), i.e. the duration between the successive tone onsets of a given player. They observed significant positive cross-correlations at lags plus and minus one between the ITIs of the two players in simple exercises for two-handed piano duets. They suggested that these correlations reflected between-player timing corrections to maintain synchrony. In their study, the player with the higher pitched melody was designated as the leader. Goebl and Palmer expected that the leader would influence the other player more strongly (greater dependence at lag plus one) than in the reverse direction (lag minus one). However, even though the mean asynchrony showed the leader did, on average, play slightly in advance of the other player, no asymmetries in ITI cross-correlations were observed, suggesting each player was correcting equally for the timing variations of the other. By contrast, asymmetries in lag-one cross-correlations of ITIs were found when one or other of the two participants was deprived of auditory feedback, and so lacked information needed to implement corrections (see also [17]).

What might be the basis for the timing corrections used by players to keep in synchrony? Vorberg and co-workers have proposed phase correction as a method for an individual performer to achieve synchrony with a periodic [18] or time varying [19] metronome click or with another performer [20]. The basic principle is that asynchrony between a tone onset and the metronome click (or between a pair of tone onsets produced by two performers), which may be described as a ‘phase’ error, is used by the performer to adjust the time interval leading up to the next tone onset. Thus, the performer shortens or lengthens the time to the next tone onset in proportion to the preceding asynchrony, so that the next tone onset and metronome click (or pair of tone onsets) are more nearly synchronous (‘in phase’).

A linear phase correction scheme for synchronization with a periodic metronome may be represented [18,19] as in equation (1.1).1.1
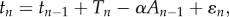
where *t_n_* and *t_n−_*_1_ are current and previous observed tone onset event times, *T_n_* is the time interval generated by an assumed internal timekeeper, *α* is the correction strength or gain, *A_n__−_*_1_ is the previous event asynchrony and *ɛ_n_* is a random error term. Elsewhere [18,19], the error term has been assumed to include timekeeper and motor components, the latter inducing negative correlation which, for the sake of simplicity, we ignore here. Whether the asynchrony is reduced completely to zero depends on the value of the gain, *α*, and hence the proportion of the preceding phase error that the performer attempts to remove. For example, if *α* = 0.5, then only half the error is removed during the interval to the subsequent tone onset.

To date, support for the linear phase correction model of synchronization (equation (1.1)) has largely been drawn from studies in which individual participants tap the index finger in time with a metronome [18,19,21,22]. There have been two approaches to estimating the correction gain in such synchronization tasks (see [23] for review). In some studies, an unpredictable perturbation of phase of the metronome (one pulse is advanced or delayed) leads participants to adjust the timing of their taps to produce damped restoration of phase, where the restoration rate provides an estimate of correction gain. In other studies, correction is evidenced by a geometric decrease with increasing lag in the asynchrony autocorrelation function (ACF) with gain estimated by the lag-one autocorrelation. However, it should be noted that some discrepancies in estimates obtained by the two methods have been reported [24]. Theoretically, it has been shown that gains within the bounds of 0 and 2 lead to stable asynchrony time series [18,19]. Gains less than one result in an overdamped asynchrony ACF (the successive terms in the ACF are negative and approach zero in geometric decreasing manner with increasing lag) and greater than one, an underdamped function (the successive ACF terms alternate between negative and positive as they approach zero). Phase perturbation studies generally reveal that correction gains are less than one except at relatively long intervals [23].

## 2. Model

Could linear phase correction serve as a model of music ensemble performance? Tapping in synchrony with a metronome differs from synchronization in music ensemble performance in various ways including: the time intervals are all equal, there is no concurrent demand on pitch or loudness (dynamics), no expressive variation is required and the adjustment process is one-way (i.e. the participant adjusts to the metronome but not vice versa). This last issue has been addressed by programming a computerized metronome to adapt to the human performer using the same principle of phase correction proposed to underpin synchronization between two people [20,25]. The linear phase correction model generates asynchrony ACFs that qualitatively match tapping data, suggesting the potential applicability of the model to ensemble synchronization in musical performance.

**Figure 1.**
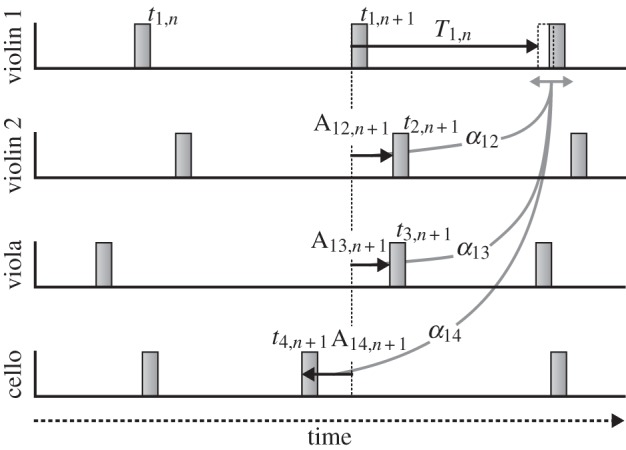
Schematic of the phase correction model for quartet synchronization from the perspective of violin 1 (i.e. player 1 in the notation of equation (2.1)).

Accordingly, in equation (2.1), we define a linear phase correction model for quartet timing using a set of linear regression equations:2.1
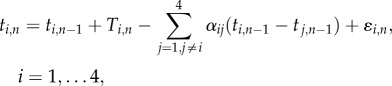
where *t_i_*_,*n*_ and *t_i_*_,*n*_ *_−_*_1_ are current and previous observed tone onset event times for player *i*, *T_i_*_,*n*_ represents the timekeeper interval, *α_ij_* refers to the correction gain applied by player *i* for the asynchrony (*t_i_*_,*n*_ *_−_*_1_ − *t_j_*_,*n*_ *_−_*_1_) with player *j*, as illustrated in figure 1, and *ɛ_i_*_,*n*_ is a random noise term identified with the assumed internal timekeeper; as with equation (1.1), we ignore motor variance.

What values of correction gain would be appropriate for four performers playing in an ensemble? In duet performance, it has been shown that, for stable performance, the sum of gains should be bounded between 0 and 2 [20]. Thus, analysis of the two-person synchronization model (*N* = 2) revealed that the asynchrony variance diverges unless 0 < *α*_12_ + *α*_21_ < 2, yielding an overdamped ACF only if *α*_12_ + *α*_21_ ≤ 1. This analysis also showed that asynchrony variance is minimized if *α*_12_ + *α*_21_ = 1, assuming, as for equation (2.1), that motor variance is negligible.

**Figure 2.**
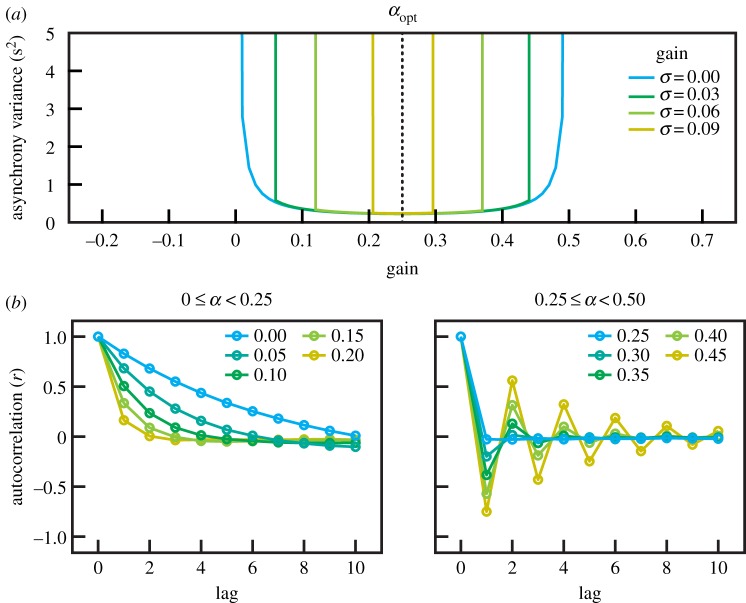
Characterization of tone asynchrony variance and ACF. (*a*) Changes in the simulated asynchrony variability as a function of the average correction gain across the ‘quartet’. Dotted line indicates where the s.d. of asynchrony was the smallest. (*b*) Asynchrony ACF averaged across all pairs of ‘players’ for the case *σ* = 0. As noted in the proof given in appendix B, the overdamped form of the asynchrony ACF with gain less than 0.25 changes to underdamped (oscillatory) for gain greater than 0.25, with the ACF being critically damped when gain equals 0.25.

We hypothesized that the condition for stable synchronization might extend to larger groups, *N* > 2. That is, the average gain across individuals might be bounded by 0 and 2/*N*, and the transition between overdamped and underdamped ACF might occur at 1/*N*, which, in a quartet would correspond to an average gain of 0.25. In appendix A, we prove that stability of the linear phase correction model of ensemble timing requires a gain between 0 and 2/*N*, assuming all gains are equal, and that, within this range, a gain of 1/*N* minimizes asynchrony variance (figure 2*a*). That is to say, as group size increases, optimal gain decreases. In appendix B, the form of the asynchrony ACF is shown to be overdamped, critically damped or underdamped when gain is respectively less than, equal to or greater than 1/*N* (figure 2*b*).

The proof that a gain of 1/*N* is optimal, in terms of minimizing asynchrony variance in ensemble timing, assumes all gains are equal. We ran computer simulations to determine whether the case treated in the proof extends to unequal gains. A sequence of 48 tone onset times with period of 190 ms for each member of a virtual ‘quartet’ was generated using equation (2.1) with initial tone onset time selected randomly from a normal distribution (*μ* = 0, *σ* = 15 ms). Normally distributed (timekeeper) noise was also added to each subsequent tone onset time from the same distribution. All 12 correction gains between all pairs of ‘players’ were set equal and varied from −0.25 to +0.75 in order to characterize the asynchrony across tone onsets in terms of standard deviation (s.d.) and between-player asynchrony ACFs. The whole iteration was repeated 1000 times, and the results were averaged across each gain, for estimates of s.d., and across lags and gains, for estimates of the asynchrony ACF. In the baseline condition where all gains were set equal (*α* = 0), the stability bounds (sharply rising asynchrony variance) were observed at gains of 0 and 0.5 (figure 2*a*). Then, we randomly varied the 12 gains, describing the correction applied by each player to asynchronies with the other three players. We systematically changed the s.d. of the gains across pairs of players to 0.03, 0.09 and 0.12. As the gain s.d. increased, the stability region progressively decreased. However, the minimum asynchrony variance was obtained at approximately 0.25 in each case, supporting our contention that a gain of 1/*N* is optimal even with unequal gains.

The present study sought support for the first-order linear phase correction model embodied in equation (2.1) in two professional string quartets treated as two separate case studies. The quartets were asked to play a 48-note excerpt from Haydn's quartet Op. 74 no. 1 fifteen times, with individual players encouraged to introduce unrehearsed, different intentional timing variations on each trial. Our model-based aims were (i) to use an iterative least-squares fitting procedure to determine whether the phase correction model affords a stable set of between-player gain estimates, (ii) to assess how good is the fit of the model to the observed data compared with the fit of the model to a dataset which is identical in content but whose order is randomized (so that any time series dependence is disrupted) and (iii) to ascertain whether the residuals, after fitting the first-order model to the observed data, are further reduced by adding a second-order correction term.

Assuming the model respected these aims, we were then interested to pursue two further challenges: (iv) to establish whether correction gain estimates approach the value of 0.25, which is optimal in minimizing asynchrony variance and (v) to discover whether the correction gains are equal across all the quartet members or whether, for example, the leader's gain is less than that of the others. On the last point, results from two-person synchronization finger tapping suggest that the gain is fixed and does not vary under normal conditions [20,25]. However, it is not clear whether this would apply to ensemble musical performance. In chamber music, one player, often the first violinist (violin 1) with the melodic line, commonly takes the role of lead [26], which may pass with the melodic line to other players as indicated by the score [12]. Under linear phase correction, asymmetry in the degree of correction between the lead and the other players might therefore be expected. Thus, if violin 1 has the melodic lead, then stronger correction by the other players (violin 2, viola, cello) for their asynchronies with respect to the lead might be expected with weaker correction by the lead for his or her asynchronies with the other players. However, the findings of fixed gain in tapping with an adaptive metronome [25] and symmetric lag-one cross-correlations in ITIs produced by piano duets [16] (consistent with equal gains in the case of first-order phase correction) suggest instead that there might be similar levels of correction between all members of a quartet.

## 3. Material and methods

### 3.1. Participants

Two professional string quartets whose repertoires span both modern and classical traditions, participated in the study. Quartet A, comprising two male and two female members, is based in Germany, with founder members having played together for 17 years at the time of recording. Quartet B, also with two male and two female members, is based in the UK, its members having played together for 10 years. The high international standard of the two quartets is evidenced by their separate tallies of 60 or more concerts in the year prior to data collection, together with five international performance prizes and at least three CDs of their recordings for each quartet since formation.

### 3.2. Apparatus

Audio data were recorded from each instrument using an omnidirectional miniature condenser microphone (model 4061s, DPA Microphones A/S, Allerød, Denmark) attached under the strings between bridge and tailpiece using a rubber mount (MHA6001, DPA). The microphone signals were sampled with a sound card (Model 8Pre, MOTU, MA, USA) at 41 kHz, and separately streamed and saved within Logic Studio Pro running on a MAC desktop PC (Apple, CA, USA). After recordings were completed, the audio data were formatted to uncompressed WAV files and analysed in Matlab (MathWorks, MA, USA).

### 3.3. Procedure

**Figure 3.**
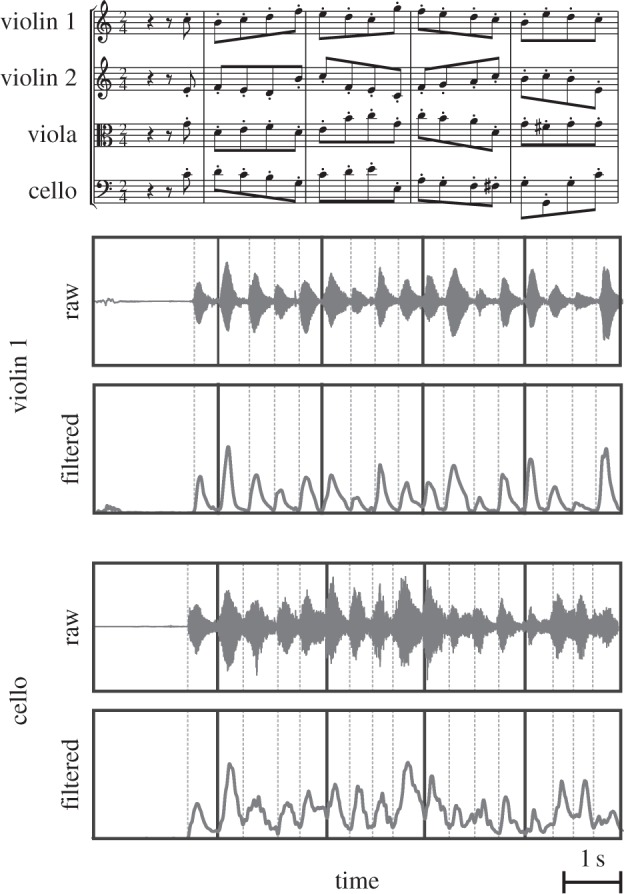
The first five bars of the excerpt from the fourth movement of Joseph Haydn Op. 74 no. 1 (above) with two channels of the audio recording from the microphones attached to violin 1 and cello (below) of quartet A in raw and filtered (rectified then low-pass filtered) forms, from which times of tone onset were determined (vertical lines).

The quartets were seated in a semi-circle of radius 2 m (quartet A) or a circle of radius 1.5 m (quartet B) measured to the front legs of the players’ chairs, which were placed in the sequence: violin 1, violin 2, viola and cello. Players faced the centre of the circle. They were able to see each other, so that visual as well as auditory cues were available to assist synchronization. Players had the sheet music for their instrument on a stand in front of them. The players were asked to play a short excerpt from the string quartet Op. 74 no. 1 (fourth movement, bars 13–24) by Joseph Haydn. This excerpt, in which violin 1 has the melody, is useful for the study of synchronization in that all four instruments are scored in rhythmic unison (homophony) to play a series of 48 eighth notes at a steady tempo (i.e. with equal ITIs), with just violin 1 breaking the pattern at note 44 with an embellishment of four sixteenth notes (see figure 3 and the electronic supplementary material, figure S1). Each quartet repeated the excerpt 15 times. The players were requested to vary their expressive phrasing, without overt verbal direction or rehearsal, over successive performances. It was expected that this would result in variation in between-individual asynchronies, necessitating corrective adjustments to preserve ensemble. We applied the linear phase correction model to all tones in the two quartets treated as two separate case studies.

### 3.4. Statistical methods

In order to determine the level of correction used by individual players, we applied the phase correction model to the ITIs derived from the multichannel audio recordings of the two quartets. The individual instrument sound recordings were rectified and then smoothed using a 50 Hz bi-directional second-order Butterworth low-pass filter [27]. Local maxima of the signal corresponding to successive tones were detected, and tone onset event times were determined using an adaptive threshold applied to the ‘valley’ preceding each maximum. This tone onset event detection method was visually cross-validated with a spectrogram of the raw signal.

Estimates of the 12 gain parameters (three correction terms for each of the four players) were simultaneously obtained by subjecting the event time data to the following asynchrony model derived from equation (2.1):3.1
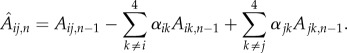


The asynchronies between player *i* and *j* were adjusted by the products of the asynchronies and correction gains of *i* and *j* with respect to all the other players *k* (yielding a total of 12 sets of asynchronies at event *n* from the observed asynchronies at the previous event, *n −* 1). The underlying assumption in this approach is that variability contributed by the assumed timekeeper is negligible to first approximation, which expresses our goal of accounting for the stochastic structure of the asynchronies in terms of error correction processes. The set of 12 gains which minimized the total variance of the differences between the predicted and observed asynchronies, 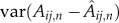, was obtained for each trial, using multivariate iterative fitting (*fminsearch* function in Matlab) where all gain parameters were constrained between 0 and 1. The averages of the gain parameters and cross-trial s.d.s are reported in the Results section.

## 4. Results

In order to give a general sense of the timing of each quartet and for comparability with previous studies [16,17], we first present statistics on ITIs. We then turn to analyse the asynchronies between each pair of players in terms of the first-order phase correction model. It should be noted that the analyses of ITIs and asynchronies were based on slightly different numbers of tones. In the case of the ITIs, we discarded tones 45 and 47 of violin 1, so that all ITIs would nominally be eighth notes. In the case of the asynchronies, we discarded the first asynchrony (because there was no previous asynchrony to serve as referent for correction) and asynchronies for tones after tone 45 (because the violin 1 ornament was difficult to score reliably in terms of tone onsets, and the phase correction model does not apply to tones that are not scored as simultaneous across all four players).

The mean ITI between the 48 tone onsets averaged over the 15 repetitions was 191.5 ms (s.d. 25.0 ms) for quartet A and 191.8 ms (s.d. 16.7 ms) for quartet B. The average s.d. of the ITIs was 32.5 ms (s.d. 8.4 ms) and 24.5 ms (s.d. 6.9 ms) for the two quartets. This variability reflects differences in tempo across trials and variation in tempo within and between bars owing to the players’ interpretations of the music's metre or ‘groove’.

**Figure 4.**
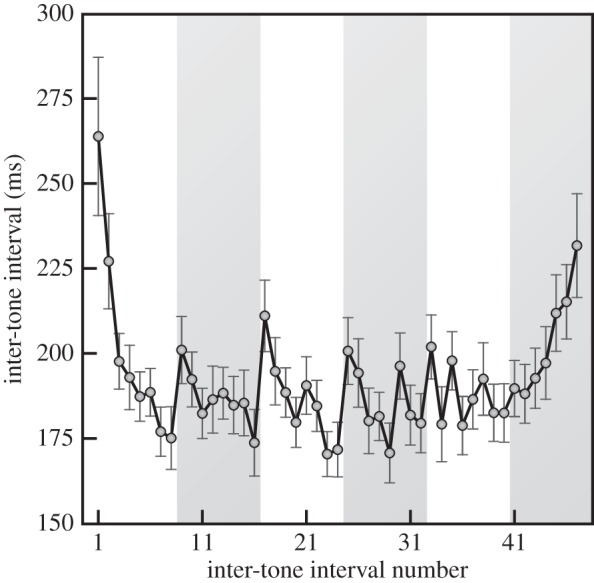
Mean interval between tone onsets as a function of tone position averaged across four players and 15 repetitions (alternate white and grey bars indicate two-bar groups. Error bars show ±1 standard error (s.e.) for the 15 replications. Intervals 45 and 46 exclude violin 1. Data from quartet A).

Figure 4 shows that ITIs in quartet A varied systematically in two-bar groups (shown alternately in white and grey in the figure), with an *accelerando* (speeding up) after a lengthened ITI (or slight pause) before the first downbeat in each group. In addition, there is a *ritardando* (slowing down) in the last two-bar group at the end of the excerpt. A similar but less pronounced patterning was evident in the ITIs of quartet B (not shown). With the ITIs adjusted to remove tempo and metre effects, the average s.d. of the ITIs was 26.6 ms (s.d. 6.1 ms) for quartet A and 17.8 ms (s.d. 3.6 ms) for quartet B.

**Figure 5.**
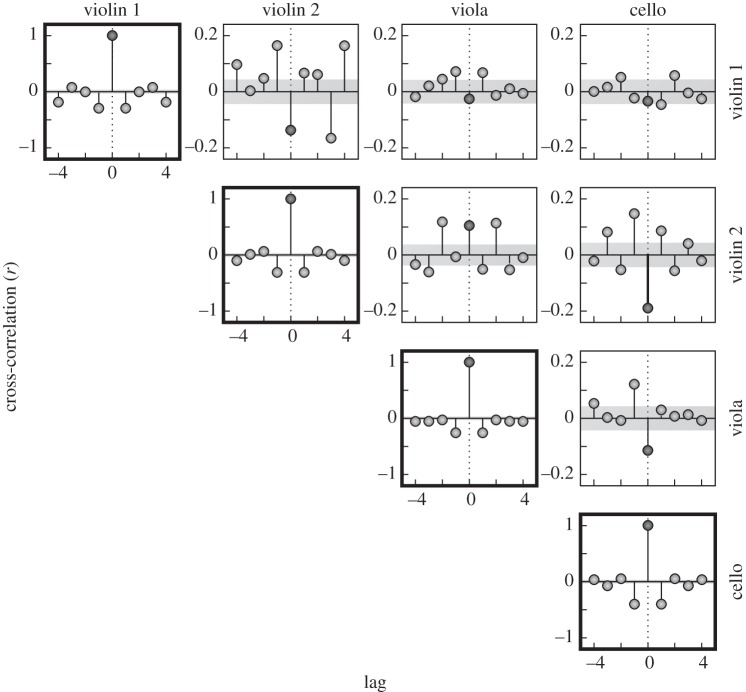
Autocorrelation (diagonal) and cross-correlation (off-diagonal) functions of the inter-tone intervals (after removal of tempo and metre effects evident in figure 4) averaged across 15 repetitions of the excerpt. (Grey region indicates ±1 s.e. of the mean; numerically small and so not visible in the case of the autocorrelation functions on the diagonal. Data from quartet A.)

The average autocorrelation and cross-correlation functions (lags from −3 to +3) of the adjusted ITIs for each instrument are shown in figure 5 for quartet A. Autocorrelations (on the diagonal) are generally negative at lag one and zero for higher lags. The negative lag-one value is indicative of event time variability, which may reflect player motor variability [28], measurement error or a combination of both. The (off-diagonal) cross-correlation functions tend to positive values at lags −1 and +1 contrasting with negative cross-correlation at lag zero (violin 1 with violin 2 and viola, violin 2 and viola with cello).

The remainder of our analysis focuses on the asynchronies between players from tones 2 to 45. The asynchronies are unaffected by ITI adjustment for tempo. The asynchronies at tone 1 were excluded from the analysis, because there was no preceding asynchrony to which equation (3.1) could be applied, and also because, in the absence of preceding notes, it seems plausible to suppose that the basis for players’ synchronization of the first note would have been different from the following notes. The tones following tone 45 were discarded because of the exceptional 16th notes in the violin 1 part. The overall average asynchrony of violin 2, viola and cello relative to violin 1 was −11.7 ms (s.d. 3.1 ms) for quartet A and −3.2 ms (s.d. 1.6 ms) for quartet B where the negative asynchrony indicates that violin 1 leads. The average asynchrony s.d. of violin 2, viola and cello relative to violin 1 (averaged across the 15 replications) was 28.3 ms (s.d. 3.2 ms) for quartet A and 24.4 ms (s.d. 5.1 ms) for quartet B.

The results of fitting the model in equation (3.1) are shown in table 1. The average percentage of variability accounted for (reduction in variance after fitting the model relative to the variance without the model) was 33.8% (s.d. 16.1%) for quartet A and 14.1% (s.d. 7.0%) for quartet B. These reductions in variance contrast with decreases of 10.4% and 3.9% obtained for quartets A and B, respectively, when the 12 sets of asynchronies for each quartet were randomly reordered and subjected to the same model fitting procedure.

**Table 1.** Average (and s.d. over 15 trials) of the variance (ms^2^) of the raw asynchrony data and the residuals for the best fit gain (*α* = fitted) and percentage reduction for the first-order phase correction model.

quartet	raw	*α* = fitted	% reduction
A	1230.9 (548.8)	767.0 (263.3)	33.8 (16.1)
B	760.3 (194.3)	649.8 (151.6)	14.1 (7.0)

**Figure 6.**
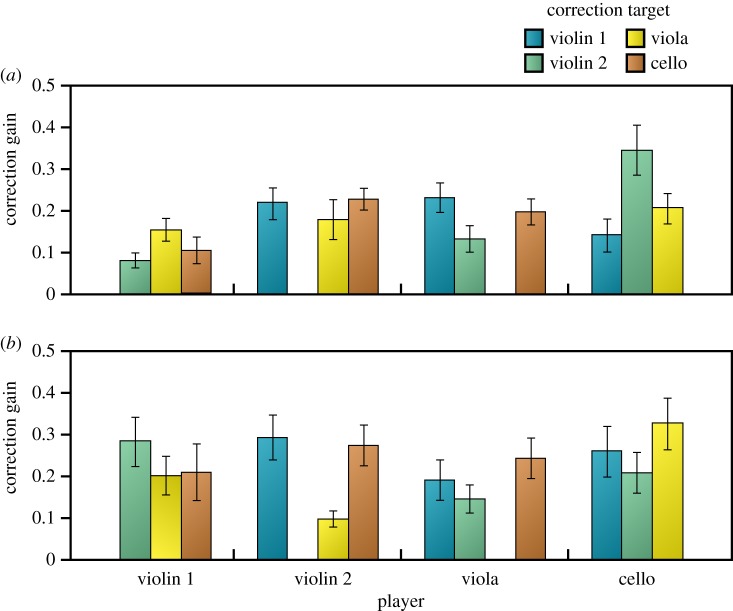
Correction gains for all pair-wise combinations of players in quartet A (*a*) and B (*b*) averaged across 15 repetitions of the excerpt (±1 s.e.).

Figure 6 shows estimates of correction gain in the first-order phase correction model for each player in each of the quartets. The overall average gain for quartet A was 0.185 (s.d. 0.020), and for quartet B, it was 0.227 (s.d. 0.035). These values were reliably less (*p* < 0.005) than the optimal gain of 0.25 (see section Introduction). Gain values for violin 1 were consistently low in quartet A 0.113 (s.d. 0.054) compared with the other players, 0.208 (s.d. 0.024). The gains for each of the other three players show that they adjusted most strongly to violin 1 and less strongly to the other two players. This was not the case for quartet B where the average gain for violin 1, 0.231 (s.d. 0.075), was not reliably different from the other players’ gains, 0.226 (s.d. 0.045). In both quartets, cello adjusted more strongly to the other players than vice versa (quartet A, 0.230 (s.d. 0.076) versus 0.169 (s.d. 0.028); quartet B, 0.263 (s.d. 0.075) versus 0.215 (s.d. 0.049). Gain estimates were stable within each quartet in that the profile of gains across the players was similar for the first (eight trials) and second half (seven trials) of the block of trials (quartet A, *r* = 0.61, *p* = 0.081; quartet B, *r* = 0.87, *p* < 0.005).

The average ITI of 191 ms might appear to leave relatively little time after each asynchrony to implement correction before the next tone is played and this led us to ask whether correction might be reduced or partly deferred to the following tone (second-order correction). In synchronization tapping using left and right index fingers in alternation, Repp [29] found that phase correction decreased as the inter-tap interval was reduced from 300 to 100 ms. To check on possible similar limitations on implementing first-order correction in our data, we tested for positive correlation between gain and mean ITI for each gain for each player in each quartet for each trial. Separately, we also applied a second-order model to the residuals after fitting the first-order model. The average correlations between gain and mean ITI across gain, player and trial were −0.11 (s.d. 0.26) for quartet A and 0.02 (s.d. 0.24) for quartet B, which were not reliably different from zero. When the second-order correction model was applied to the residuals after fitting the first-order model, small non-significant further reductions of 1.5% and 2.1% in variance were achieved (to 34.8 ± 15.7% for quartet A and to 16.7 ± 7.3% for quartet B). We conclude that, despite the short inter-tone intervals, the correction applied by the quartets was effectively first-order.

In summary, the first-order phase correction model applied to all tones resulted in an average gain estimate that was near, but slightly below, the value of 0.25 which would be optimal in terms of minimizing asynchrony variability. There were individual differences between players (e.g. cellos more dependent on the others than vice versa) and between quartets (violin 1 in quartet A but not B less dependent on the others than vice versa) in the degree of correction. There was no evidence that relatively short ITIs resulted in second-order correction.

## 5. Discussion

Where notes are scored to be played together, performers in a chamber music quartet normally seek to synchronize their tone onsets, possibly with the melodic lead slightly ahead of the others for acoustic emphasis. In this study, we encouraged quartet members to introduce timing variations illustrative of the range of expressive performance as they repeatedly played a short 12-bar excerpt from the fourth movement of Haydn Op. 74 no. 1. Because these variations were unrehearsed and the quartet members were asked not to exchange explicit verbal cues, and given, in addition, unintended timing variability, we expected that there would be fluctuating asynchronies between players which they would seek to compensate. Both quartets exhibited variability in the asynchronies which was similar in magnitude to that reported by Rasch [30], who was first to document variability in ensemble playing from sound recordings of wind and string trios. Moreover, the two quartets exhibited similar mean and variability of ITIs, giving us some confidence that the players met the request to vary their expressive phrasing in a manner resulting in variation similar to that which might be expected across live performances.

Given variability in asynchrony, the quartets must necessarily have made timing adjustments to maintain synchrony of the ensemble. The aims of the study may therefore be summarized: first, to determine whether the linear phase correction model affords a stable set of between-player gain estimates and to assess the model's goodness of fit. Second, to establish whether, on average, estimates of correction gain approach the optimal value of 0.25 and to discover whether they are equal across all members of each quartet.

We applied a first-order linear phase correction model of synchronization to the players’ tone onset times in order to estimate the correction gains each player used in adjusting to the asynchronies with each of the other players. The model provided small positive estimates of correction gain accounting, on average, for 23.9% of the asynchrony variance. These gains were greater than zero and consistent across trials, with the average gain for each quartet being slightly less than the value of 0.25 which, in terms of the model, was predicted as optimal for the reduction of the variability of asynchrony. These results give confidence to our extension of the first-order linear phase correction model, originally proposed by Vorberg and co-workers [18,19] for tapping with a periodic metronome, to musical performance by string quartets. The results also provide quantitative support for Goebl & Palmer's [16] suggestion that synchronization in musical ensemble involves reciprocal correction between players.

The average correction gains for the two quartets were generally slightly smaller than the optimal value of 0.25. One possible reason for gain values less than 0.25 is that, contrary to our simplifying assumption, there is appreciable motor variance. Motor variance jitters the timing of each tone producing asynchrony variance which, unlike variance in the assumed timekeeper, does not affect the phase of the following tones. One effect of motor variance is to introduce negative lag one autocorrelations in the ITIs [28], of which there was some evidence in figure 5. Analysis of the equal-gain version of the model that includes motor variance shows that, as motor variance increases, the degree of correction should reduce. In the present case, the gain was some 20% less than the predicted optimal value of 0.25. Derivations based on the equal-gain model, as well as simulations of the more general model, suggest motor variance amounts to some 20% of the total variance of asynchrony. Evidently, this would be a useful area for future evaluations of our proposed model and might contribute to reducing the unexplained variance.

Fitting a second-order model to the residuals after fitting the first-order model accounted for only slightly more of the variance than the first-order model. Moreover, there was no reliable positive correlation across trials between gain estimates and ITI. Taken together, this is evidence against the possibility that the relatively short time-interval between asynchrony and the next tone might have limited the gain or tended to extend correction over two tones. This may be seen as consistent with Repp [29] who, in a study of tapping, noted that, at very fast response rates, e.g. 100 ms inter-tap interval, lack of time in which to effect correction led to low estimates of phase correction, whereas for longer tap intervals around 200 ms, phase correction was almost as strong as at 300 ms.

Simulation results (see the electronic supplementary material, figure S2) show that, without correction, the average asynchrony between players increases linearly with successive tones, whereas even the small degree of correction observed is sufficient to produce asymptotically stable levels of asynchrony. Simulation also shows that larger levels of gain applied between all pairs of players result in asymptotically stable asynchrony. However, the ACFs reveal a tendency to underdamped, oscillatory behaviour and such oscillation, if perceptible, might be experienced as musically unsatisfactory. We are currently conducting experiments to determine to what extent different patterns of correction gain between players in an ensemble are perceived by the listener.

In quartet A, the gains applied by violin 1 to her asynchrony with the other players tended to be smaller than those applied by the other players to their asynchronies with violin 1. This pattern of gains, combined with a small but reliable phase advance of violin 1 over the other instruments, may be seen as consistent with the role of leader ascribed to violin 1 when, as in the present excerpt, she has the melody. Thus, it was the supporting players, rather than the leader, who adjusted their timing to maintain ensemble. In quartet B, the gains of violin 1 with respect to the other players were the same as those used by the other players with respect to violin 1 and there was minimal temporal lead of violin 1 over the other players. This difference between quartets suggests a difference in ‘strategy’, with quartet A agreeing on violin 1 leading and quartet B with more democratic ensemble. Both may be seen as musically valid in the context of the movement's opening 12 measures, which immediately precede the excerpt used in this study and in which violin 1 unquestionably carries the tune. Our excerpt (starting with the upbeat to measure 13) has violin 1 now losing its soloistic articulation and falling into a *tutti staccato assai*, though with the same pitches (tune) as before. Quartet A perhaps interpreted the unchanged tune as meaning violin 1 should still play as solo, whereas quartet B sought to even out the ensemble hierarchy (rhythmic unison, *tutti staccato*) in spite of the repeat of the tune. It is clear that our analysis indicates the potential for further investigations with other quartets and other musical examples.

Although differences were observed in the violin 1 gains, a similarity between the quartets was found in the gains of the cellists relative to the other players. Thus, both quartets showed larger gains by the cellist compared with the other players’ gains with respect to the cellist. This finding is intriguing, as it might be seen as going against the perception in music of the role of the cello as providing a rhythmic basis for small ensembles. Further research is needed to determine whether this is a general phenomenon or whether it reflects a specific aspect of the chosen musical excerpt. For example, the professional cellist among us (A.B.) observes that the octave, cross-string leaps in the cello part are a technical challenge that may have resulted in an increase in ‘catching up’ behaviour. The other instruments also have octave leaps, but an octave on a violin as well as also on the viola does not involve the distances, and therefore the hand position changes, that the cellist must endure. Some support for the greater relative difficulty of the cello part was found in greater variance in ITI of the cello player (quartet A, 1372.7 ± 559.0 ms; quartet B, 1539.1 ± 511.3 ms) compared with the other players (quartet A, 882.7 ± 711.3 ms; quartet B, 535.4 ± 278.0 ms), with stronger negative lag-one autocorrelation (quartet A, *r* = −0.40 ± 0.18 versus −0.35 ± 0.10; quartet B, *r* = −0.50 ± 0.12 versus −0.46 ± 0.08).

Given evidence of phase correction between players in maintaining ensemble, it is interesting to ask what cues to timing the players might have been using. The model we used in our time series approach was based on asynchronies, with tones defined by their acoustic onset. However, there are other cues that players might use when synchronizing with one another. In the acoustic domain, there are plausible alternatives to tone onset such as spectral flux, tone peak amplitude or some combination of features, which, together, define the perceptual centre of a tone [31]. It is also possible that players use cues from other modalities. Thus, players’ bow movements, but also facial gestures and whole body sway, are all potential cues to timing [8]. Visual cues such as these might be expected to be particularly important at entry points, such as the first note of the passage. In the Goebl & Palmer [16] study, duetting pianists either received full auditory feedback, one-way feedback (leaders heard themselves, whereas followers heard both parts), or self-feedback only. Motion analysis showed that leaders raised fingers higher and head movements became more synchronized as auditory feedback was reduced which suggests that visual cues become more important when auditory information is absent. However, under the conditions of the present study (full auditory information was available, the spatial arrangement of players made visual integration across players difficult), it seems reasonable to assume synchronization was based on auditory rather than on visual cues except for the first tone (which was omitted from the time series analysis) when all players might have been expected to watch violin 1.

How realistic is the paradigm we used for quartet playing in general? Our method of asking players to introduce expressive variation might seem somewhat contrived. However, expressive variation is central to expert creative performance, as noted in the Introduction, and as recently described by Seddon & Biasutti [32] who refer to it as empathetic attunement. Thus, ‘musicians seemed to respond to each other in an atmosphere of risk taking and challenge, which extended their joint creativity. They took risks with musical phrasing, timing, and dynamics in that they challenged each other's musical creativity’. Such empathetic attunement was seen as contrasting with sympathetic attunement with ‘predictable performance providing musical cohesion without creative risk through adhering to previously rehearsed interpretations’. In this respect, it is interesting to observe the suggestion by Yamamoto & Miyake [33] that cooperative performance may extend beyond the music to physiological measures such as heart rate and breathing, and the recent findings of between-player EEG coherence when playing guitar in duets [34].

In summary, we have subjected the craft of string quartet playing to time series analysis in order to investigate the hypothesis that ensemble synchronization is achieved through linear phase correction. In two different quartets playing an excerpt from Haydn, we showed average asynchrony correction gains approaching the value of 0.25 that was predicted by our model of optimal four-person synchronization. However, the pattern of individual players’ gains within a quartet differed between the two ensembles, with one quartet (B) more symmetrical in gain values than the other. One reason for this could be different, though equally valid, musical interpretations of the same excerpt. For the future, we envisage such analyses applied to other musical passages, to determine whether assignment of the melody to a different player/instrument changes the functional leadership and so introduces an asymmetry in correction gains, as seen in violin 1 of quartet A. It will also be important to evaluate the performance of other quartets with different styles or skill levels; quartets A and B are internationally acclaimed with very extensive experience, so it will be important to evaluate less expert groups and to track the effects of skill development. We therefore propose that time series modelling of the kind outlined in this paper is a powerful means of revealing the nature and expertise of cooperative timing in small musical ensembles.

## Figures and Tables

**Figure 7.**
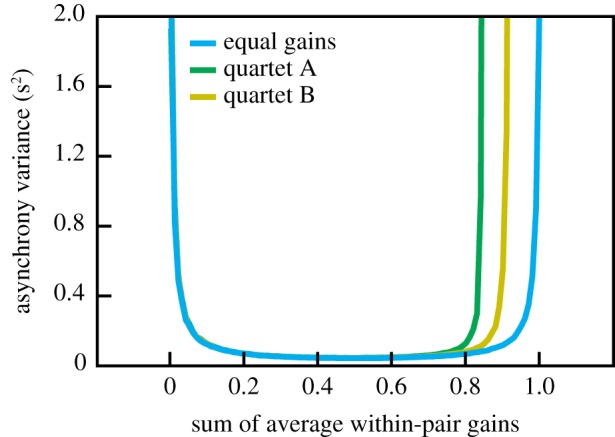
Monte Carlo study of the quartet asynchrony variance when the correction gains between players were varied. The datasets were simulated from equation (2.1) of the main text and iterated 1000 times to smooth the figure.

## Data accessibility

Event time data for the present study are presented in the electronic supplementary material.

## Appendix A. Model assumptions and proofs

For an ensemble of *N* players, we assume that the tone onset events of player *i* are timed from his previous tone onset after a delay, *T_i_*_,*n*_, which is adjusted for the asynchronies from all players:

A1

Note that the sum contains the zero term, *α_ii_*(*t_i_*_,*n*_ − *t_i_*_,*n*_), which simplifies the notation as well as the derivation below. Assuming identical gains across all pairs of player, this simplifies to

The asynchrony of player *i* with respect to player *j*, *A_ji_*_,*n*_, is defined by *t_i_*_,*n*_ − *t_j_*_,*n*_. Thus,

A2

where the difference variables, *D_ij_*_,*n*_ ≡ *T_i_*_,*n*_ − *T_j_*_,*n*_, are uncorrelated with the asynchronies, *A_ij_*_,*n*_. Now, consider the variances of the asynchronies between any two players *i* and *j*. By the recursion, we have

If the process is stationary asymptotically, then the variances must tend to limit var(*A_ij_*) ≡ lim*_n→∞_*var(*A_ij_*). Asymptotic stationarity thus implies that  , which has solution

A3

The asymptotic variances are non-negative and finite only if 0 < *Nα* < 2. For ensemble performance to remain stable, the players must adjust their gains such that the total sum remains within these bounds. Note that asynchrony variance is smallest, i.e. optimal synchronization is achieved if and only if *Nα* = 1, i.e. if *α* = 1/*N*.

For unequal rates, the corresponding analysis becomes much more involved. Our prediction is that stable ensemble performance cannot be achieved unless the summed *averaged rate* remains bounded, i.e.

A4

and that overall asynchrony variance is smallest if the sum equals unity. Note that individual players may well violate these bounds, e.g. compensate for or even amplify asynchronies with particular players much more strongly, without jeopardizing stable ensemble performance, provided the total rate sum remains bounded.

To estimate the effect of unequal gains, the set of 12 correction gains estimated from the real quartets’ performance was normalized, so that the sum of mutual gains (*α_ij_* + *α_ji_*), averaged across all pairings, was varied to shift between 0 and 2, with coefficient *C* defined by:

A5

As expected, the gains estimated from both quartets exhibited smaller stable boundaries than when the gains were equal across pairs of players (figure 7). The reduction of the higher stable boundary was smaller for quartet B owing to a smaller variability in the estimated gains (*σ* = 0.065) compared with quartet A (*σ* = 0.070)

## Appendix B. Form of the autocorrelation function

From the asymptotic asynchrony variance [3], it is straightforward to derive the predicted dependence structure of the asynchronies between pairs of players as a function of their lag, as summarized by the corresponding autocovariance and autocorrelation functions (ACFs). We define

B1

By equation (B 2), we have

and thus

because current asynchronies are independent of later inter-response intervals.

For *k* = 1, this yields

and thus, asymptotically,

and by induction,

B2

The ACF of the asynchronies follows from (B2) after normalizing by the variance, var(*A_ij_*).

For any two players *i* and *j*, the degree of dependence between their synchronization errors on the current tone diminishes with lag *k*. As shown above, the process becomes stationary asymptotically only if 0 < *Nα* < 2. Note that the ACF and the autocovariance function show a *monotonic* decrease with lag if 0 < *Nα* < 1, but an *oscillatory* decrease if 1 < *Nα* < 2; asynchronies are uncorrelated if *α* = 1/*N*.

Figure 2 illustrates this for four players with various gain values.
